# Ginger Alleviates DSS-Induced Ulcerative Colitis Severity by Improving the Diversity and Function of Gut Microbiota

**DOI:** 10.3389/fphar.2021.632569

**Published:** 2021-02-22

**Authors:** Shanshan Guo, Wenye Geng, Shan Chen, Li Wang, Xuli Rong, Shuocun Wang, Tingfang Wang, Liyan Xiong, Jinghua Huang, Xiaobin Pang, Yiming Lu

**Affiliations:** ^1^ School of Medicine, Shanghai University, Shanghai, China; ^2^ Eight Plus One Pharmaceutical Co., Ltd, Guilin, China; ^3^ Institutes of Integrative Medicine, Fudan University, Shanghai, China; ^4^ School of Pharmacy, Henan University, Kaifeng, China; ^5^ Department of Critical Care Medicine, Shanghai Tenth People’s Hospital, School of Medicine, Tongji University, Shanghai, China

**Keywords:** colitis, ginger, 16S rRNA, intestinal microbiota, dextran sodium sulfate

## Abstract

The effects of ginger on gastrointestinal disorders such as ulcerative colitis have been widely investigated using experimental models; however, the mechanisms underlying its therapeutic actions are still unknown. In this study, we investigated the correlation between the therapeutic effects of ginger and the regulation of the gut microbiota. We used dextran sulfate sodium (DSS) to induce colitis and found that ginger alleviated colitis-associated pathological changes and decreased the mRNA expression levels of interleukin-6 and inducible nitric oxide synthase in mice. 16s rRNA sequencing analysis of the feces samples showed that mice with colitis had an intestinal flora imbalance with lower species diversity and richness. At the phylum level, a higher abundance of pathogenic bacteria, *Proteobacteria* and *firmicutes*, were observed; at the genus level, most samples in the model group showed an increase in *Lachnospiraceae_NK4A136_group*. The overall analysis illustrated an increase in the relative abundance of *Lactobacillus_murinus*, *Lachnospiraceae_bacterium_615*, and *Ruminiclostridium_*sp*._KB18.* These increased pathogenic bacteria in model mice were decreased when treated with ginger. DSS-treated mice showed a lower abundance of *Muribaculaceae*, and ginger corrected this disorder. The bacterial community structure of the ginger group analyzed with Alpha and Beta indices was similar to that of the control group. The results also illustrated that altered intestinal microbiomes affected physiological functions and adjusted key metabolic pathways in mice. In conclusion, this research presented that ginger reduced DSS-induced colitis severity and positively regulated the intestinal microbiome. Based on the series of data in this study, we hypothesize that ginger can improve diseases by restoring the diversity and functions of the gut microbiota.

## Introduction

Ulcerative colitis (UC) is an idiopathic chronic inflammatory disease of the colonic mucosa, which starts in the rectum and usually continuously extends proximally through part of or the entire colon. The most common clinical symptoms of UC include diarrhea, abdominal pain and bloody mucoid stools (Silverberg et al., 2005).

The exact cause of UC is still unknown, but extensive research suggested that aberrant responses to environmental factors, genetic susceptibility, abnormal immune regulation, the intestinal mucosal barrier, and intestinal microecological changes contribute to the occurrence and development of UC (Heller et al., 2005; Dong et al., 2019).

The expression of *β*-defensin produced by intestinal epithelial cells to promote host defense and limit bacterial invasion has also been reported to be upregulated in colon samples of UC patients (Rahman et al., 2010). Recent years, a more popular theory indicted that dysregulation of immune responses to microbes in the gut contributed to the occurrence, progression, and changes of UC (Zheng et al., 2017). The innate and adaptive immunity of the host can prevent the invasion of harmful bacteria, tolerate the normal microflora. However, imbalance intestinal flora reduces intestinal immunity, resulting in overstimulation of intestinal mucosal immune response, finally contributing to the disease (Zhang et al., 2015).

Human gut microbiome disorder is closely related to a variety of human diseases like autism and mood disorders (Mangiola et al., 2016), as well as obesity, diabetes and cardiovascular diseases (Hills et al., 2019). Series of researches proved that gut microbiome played an important role in the etiopathogenesis and treatment of inflammatory bowel disease (IBD) (Masoodi et al., 2019). Colonocyte metabolism regulates the content of anaerobic bacteria, and restoration of this colonocyte metabolism seems to be a novel therapeutic approach for IBD (Litvak et al., 2018). The intestinal microbiome is a diverse combination of bacteria, archaea, fungi, prokaryotes and viruses located in the intestines of all mammals. Fungi and bacteria occupy the main position, with the most abundant and diverse in the distal ileum and colon (Ley et al., 2006). Researchers established that intestinal microbiome was a strong inducer of pro-inflammatory T helper 17 cells (TH17) and regulatory T cells (Tregs) in the intestinal, which regulated type 2 responses and balanced mucosal immune responses by inducing type 3 retinoic acid-related orphan receptor-γt (RORγt) (+) Tregs cells and TH17 cells (Ohnmacht et al., 2015). The fecal microbiome of UC patients showed significantly less biodiversity than that of healthy people (Zhang and Li, 2014). A common difference between Crohn disease (CD) and UC patients was that they showed a decrease in the proportion of *Firmicutes* and a contrasting increase in that of *Proteobacteria* (Marchesi et al., 2016). *Akkermansia muciniphila*, the most abundant bacterial species in the human intestinal flora, has also been observed to decrease significantly in UC patients (Bajer et al., 2017).Various novel therapeutic approaches using prebiotic, probiotic, symbiotic and fecal microbiota transplantation (FMT) as complementary and alternative medicine has improved the condition of IBD patients (Khan et al., 2019). Probiotics can repair the damaged intestinal mucosal barrier in UC patients (Abraham and Quigley, 2017). A randomized controlled trial showed that FMT can be used to relieve the symptoms of UC patients in the short term (Narula et al., 2017).

Ginger (*Zingiber officinael*) is a perennial rhizome herb that has been globally used to treat gastrointestinal disorders such as nausea, dysentery, diarrhea and infections. Recent years, numerous studies proved that ginger exert anti-inflammatory, antioxidant, antitumor and antiulcer effects (Mohd Sahardi and Makpol, 2019; Nikkhah Bodagh et al., 2019). Ginger extract 6-gingerol has been proved to induce the apoptosis of gastric cancer cells through tumor necrosis factor (TNF)-related apoptosis-inducing ligand-(TRAIL-)(Prasad and Tyagi, 2015). The high content of 6-shogaol contributed to the antitumor activity of ginger against breast, cervical and hematological cancer *in vitro* (Sharifi-Rad et al., 2017; Mahomoodally et al., 2019). In addition to gastrointestinal disorders, ginger also alleviated fatty liver and irritable bowel syndrome (Nikkhah Bodagh et al., 2019). Ginger nanoparticles (GDNPs 2) have been shown to relieve colitis by reducing the expression of inflammatory cytokines in FVB/NJ mice colitis model (Zhang et al., 2016) while ginger exosome-like nanoparticles alleviated colitis in mice model via *Lactobacillus rhamnosus* (LGG) (Teng et al., 2018).

In this study, we elucidated the effects of ginger on intestinal microbiome in dextran sulfate sodium (DSS) induced mice. Fecal samples were collected and 16s rRNA sequencing was perform to investigate the changes in the gut microbiota.

## Materials and Methods

### Biological Materials and Reagents

Ginger powder was purchased from Fujian Longzhi Biotechnology Co., Ltd (Fujian, China), and DSS salt (reagent grade) was purchased from MP Biomedicals (Santa Ana, CA, USA). Chloroform was purchased from Sinopharm Chemical Reagent (Co., Ltd.) (Shanghai, China). Sulfasalazine (SASP) was purchased from Dalian Meilun Biotechnology Co., Ltd (Dalian, China). RNAiso Plus, TB Green® Premix Ex Taq™ II, and PrimeScript™ RT Master Mix were purchased from Takara Biomedical Technology Co. (Beijing, China). QIAamp 96 PowerFecal QIAcube HT kit was purchased from Thermo Fisher Scientific Inc (Waltham MA, USA). All other chemicals and reagents used in this study were of analytical grade.

### DSS Induced Colitis Mice Model

Male BALB/c mice (weighing 18–22 g) were purchased from the Experimental Animal Center, Second Military Medical University (Shanghai, China) and were maintained under a 12 h light-dark cycle at a temperature of 24°C. After adapting to the environment, 32 mice were divided into four groups. All animal experiments were conducted according to the Guide for the Care and Use of Laboratory Animals published by the National Institutes of Health, and the study protocol was approved by the Animal Care and Use Committee of the Shanghai University. The DSS-induced colitis mice model were established as previously described (Chassaing et al., 2014). Briefly, BALB/c mice were administered 2.5% DSS for 7 days, Weighing body weight and monitoring disease action index (DAI) daily.

After acclimatization for 3 days, mice were randomly divided into four groups as follows: 1) the control group, fed with standard rodent food and water, without any other treatment; 2) the model group provided free access to 2.5% DSS, and orally administered normal saline at 100 μL/20 g daily; 3) provided free access to 2.5% DSS and orally administered SASP at 400 mg/kg daily; 4) provided free access to 2.5% DSS and orally administered ginger at 500 mg/kg daily. The dose of ginger was determined according to the method by Teng et al. (2018), because we had used the same ginger. Body weight and DAI were noted daily.

### Sample Collection

At the end of the experiment, fresh fecal samples were collected from the mice in autoclaved Eppendorf tubes and stored at −80°C before mice were sacrificed. The length of each colon was measured and divided into three parts for real time-PCR, and histopathological studies.

### Histopathological Studies

The colon tissues were formalin-fixed, paraffin-embedded, cut into 5 μm sections, and stained with hematoxylin and eosin (H&E). Colonic histological damage was observed under a light microscope.

### RNA Extract and Real-Time PCR

Total RNA was extracted from the colon tissue using with RNAiso Plus and chloroform, and then the total RNA concentration and purity were measured using a NanoDrop 2000 spectrophotometer. cDNA was synthesized using the PrimeScript™ RT Master Mix. Real-time PCR was conducted using TB Green® Premix Ex Taq™ II, and the reaction solution contained 5 μL TB Green® Premix Ex Taq™ II, 0.5 μL of each primer (10 μM), 3 μL genomic DNA, and nuclease-free water (total volume 10 μL). The following primer sequences of *glyceraldehyde 3-phosphate dehydrogenase* (*GAPDH*), *interleukin 6* (*IL-6)* and *inducible nitric oxide synthase (iNOS)*, are presented in Table 1.

**TABLE 1:** Primer sequences of inflammatory factor.

*GAPDH*	Forward primer	5′-AGG TCG GTG TGA ACG GAT TTG-3′
	Reverse primer	5′-TGT AGA CCA TGT AGT TGA GGT-3′
*IL-6*	Forward primer	5′-CCA ATG CTC TCC TAA CAG AT-3′
	Reverse primer	5′-TGT CCA CAA ACT GAT ATG CT-3′
*iNOS*	Forward primer	5′-GCC AGT CAG GTC TCA GCA AG-3′
	Reverse primer	5′- CGC ATG CAA TGT GTG CTT GT-3′

### DNA Extraction

Total genomic DNA was extracted using QIAamp 96 PowerFecal QIA cube HT kit (Qiagen), and the integrity and concentration were measured using NanoDrop (Thermo Fisher Scientific) and 1% agarose gel electrophoresis (voltage, 120 V; electrophoresis time, 15 min).

### 16S rRNA Sequence

16S rRNA sequencing was performed by Oebiotech (Shanghai, China), the amplification of the V3V4 region (343–798) of the 16S rRNA gene was conducted with these primer pairs: 343F-5′-TACGGRAGGCAGCAG-3′, 798R-5′- AGGGTATCTAATCCT-3′. For library construction, the 30 µL PCR mixture consisted of 10–50 ng DNA template, 1 µL each of the forward and reverse PCR primers (5 pmol/μL each), 15 µL 2× Gflex PCR buffer (Takara), 0.6 μL Tks Gflex DNA polymerase (1.25 U/μL, Takara), and an appropriate volume of double distilled water (ddH2O) was added to make up the volume to 30 μL. The PCR program was as follows: initial denaturation at 94°C for 5 min, followed by 26 cycles of denaturation at 94°C for 30 s, annealing at 56°C for 30 s, extension at 72°C for 20 s, and a final extension at 72°C for 5 min.

### Bioinformatic Analysis

Raw data were analyzed in the FASTQ format, and Trimmomatic software was used to preprocess paired-end reads. After trimming, paired-end reads were assembled using FLASH software, and reads with chimeras were detected and removed using UCHIME software. Clean reads were clustered into Operational Taxonomic Unit (OTUs) with 97% similarity cutoff using Vsearch software. The representative reads were selected using QIIME and blast against Greengenes or Silva (version 132) using a PDR classifier.

### Statistical Analysis

All values are expressed as mean ± standard error of the mean (SEM). All statistical analyses were performed using GraphPad Prism. Differences between groups were analyzed using the two-tailed Student’s *t*-test, and results with *p* < 0.05 were considered statistically significant.

### Sequence Accession Numbers

The sequences generated in the present study are available through the NCBI Sequence Read Archive (accession number PRJNA680889).

## Results

### Ginger Alleviates the Symptoms and Inhibits the Inflammation in DSS Induced Mice Colitis

**FIGURE 1:**
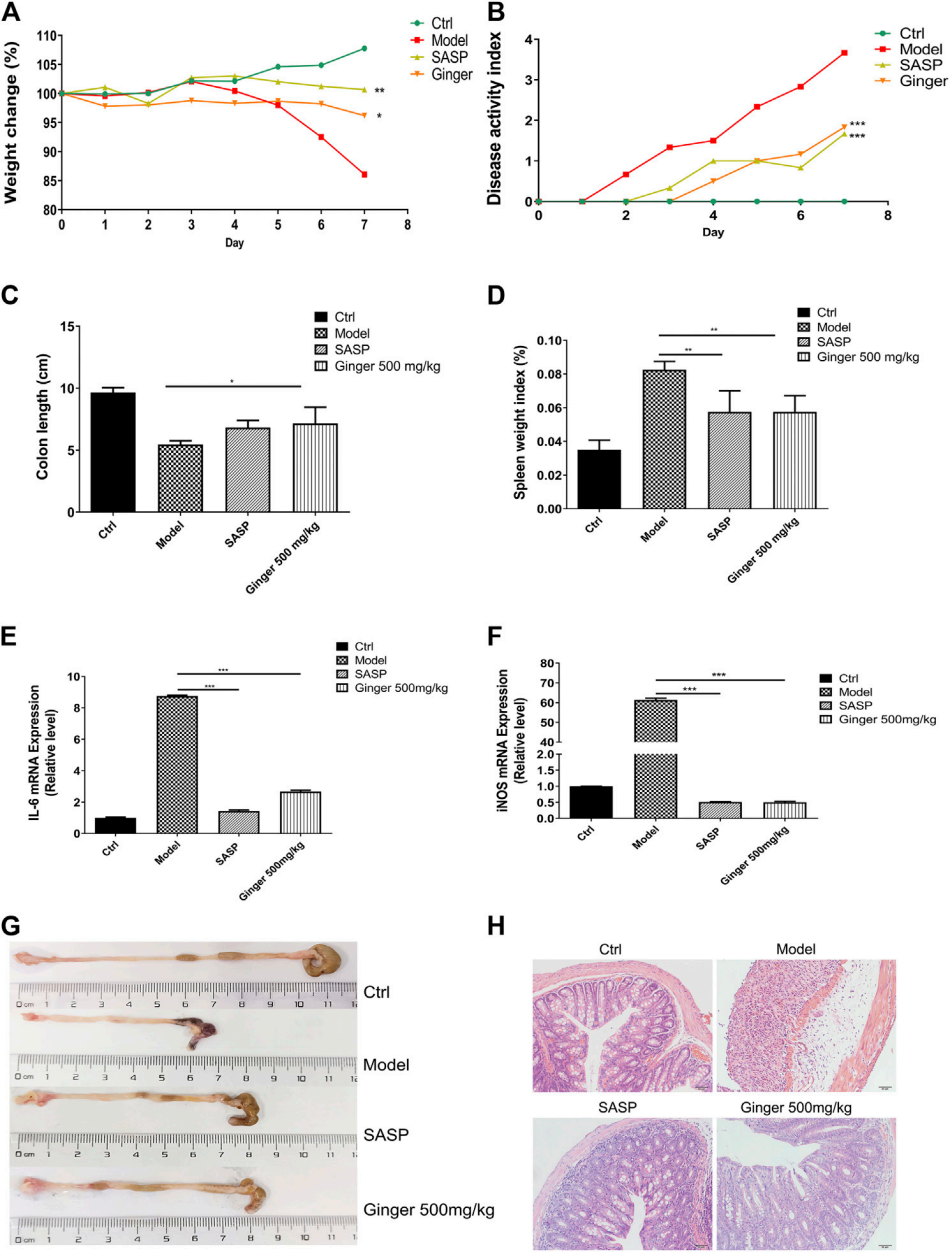
Ginger alleviates the symptoms and reduced the mRNA expression of inflammatory cytokines in DSS induced mice colitis **(A)** Body weight of mice. **(B)** Disease activity index (DAI). **(C)** Colon length. **(D)** Spleen index. **(E)** Interleukin 6 (*IL-6*) expression. **(F)** Inducible nitric oxide synthase (*iNOS*) expression. **(G)** Images of colon samples showing colon length. **(H)** Images of hematoxylin and eosin **(H and E)**-stained colon tissue samples. Magnification, ×200. Values are mean ± standard error of the mean (SEM); *p < 0.05, **p < 0.01, and ***p < 0.001 for ginger and SASP vs. model group.

As shown in Figure 1A, mice administered ginger and SASP shown a lower weight loss than the model group. The disease activity index (DAI) of mice administered Ginger and SASP were also decreased significantly (Figure 1B). Colon length shortening was less in ginger- and SASP-treated mice than the model group mice (Figure 1C). The spleen index of the ginger- and SASP-treated mice was significantly lower than the model group mice (Figure 1D).

Real-time PCR analysis revealed significantly increased mRNA levels of *IL-6* and *iNOS* in the model mice, whereas SASP and ginger treatment decreased the mRNA expression levels of these inflammatory cytokines. Ginger was less effective than SASP in decreasing the expression of *IL-6* (Figure 1E) but they were both equally effective in decreasing the expression level of *iNOS* (Figure 1F). Ginger-treated colitis mice exhibited less reduction in colon length than the model group mice (Figure 1G). Hematoxylin and eosin (H&E) staining of the colon tissues showed less severe intestinal mucosa injury (destruction of the epithelial structure and inflammatory cell infiltration) in SASP and ginger treatment groups than the model group (Figure 1H).

### Ginger Restores the Composition of Intestinal Flora in Colitis Mice

**FIGURE 2:**
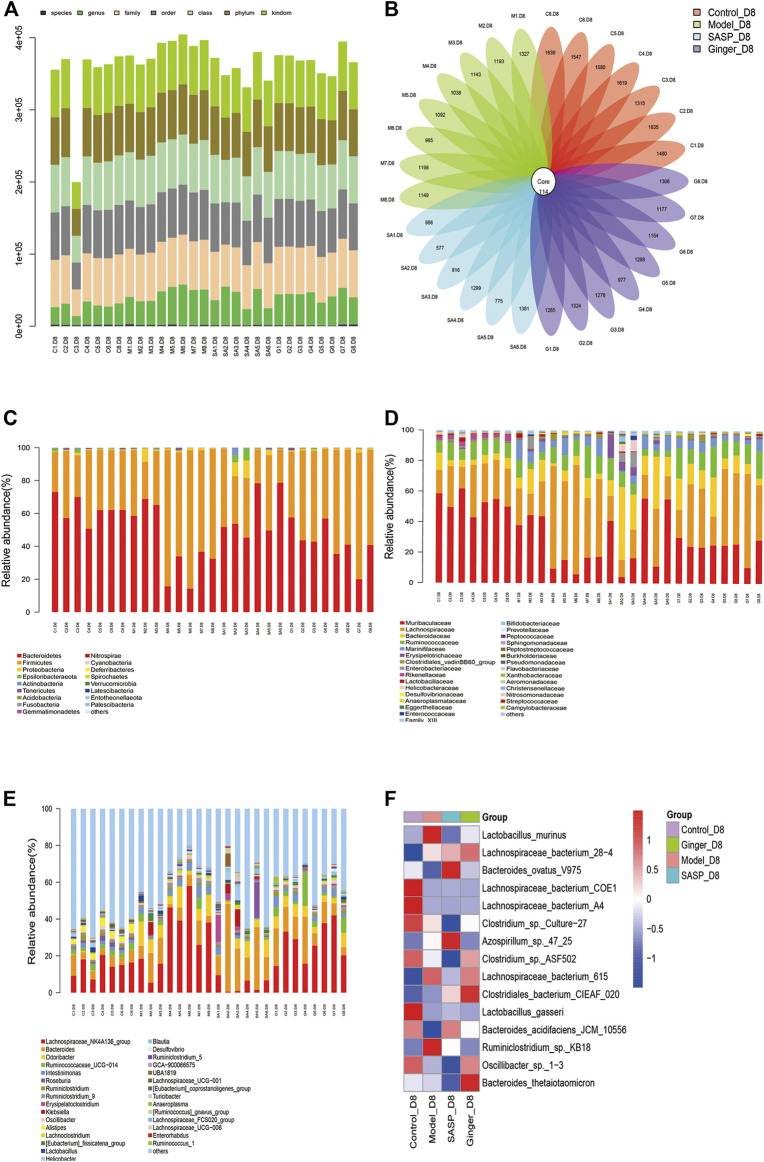
Analysis of the microbial community structure of each group **(A)** Operational taxonomic unit (OTU) level bar plots. The *X*-axis shows the name of each sample, and the *Y*-axis is the total number of tags in OTUs under class. **(B)** Flower plot analysis of the number of OTUs in each sample. The values were obtained by subtracting representative sequences from OTU counts. OTUer ring indicates samples. Values on petals were the exact OTU numbers of each sample. The center of the circle represents sequences. **(C)** Relative abundance of phyla. Each column represents a sample. Different colors indicate different bacteria in phyla. The *X*-axis represents samples, and the *Y*-axis shows relative abundance in phyla. **(D)** Relative abundance of families. Each column represents a sample. Different colors indicate different bacteria in families. The *X*-axis represents samples, and the *Y*-axis is the relative abundance of families. **(E)** Relative abundance of genus. Each column represents a sample. Different colors indicate different bacteria in genus. The *X*-axis represents samples, and the *Y*-axis is the relative abundance of classes. **(F)** Heatmaps illustrate the relative abundance of families in samples, cluster tree on the left represents the clustering of families. The above-clustering branch group represents samples from different groups. Orange and blue indicate higher and lower relative abundance, respectively.

The total number of tags in the OTU was collected to obtain the OTU level bar plot of each sample (shown in Table 2 and Figure 2A). After quality control, approximately 41,264 and 74,044 clean tags were obtained; the chimeras were removed and the valid tags obtained were used for the analysis. The average length of valid tags ranged from 405.99–417.12 bp. The number of OTUs in each sample was counted by subtracting the representative sequences from OTU counts, and it ranged from 577–1835 bp (Figure 2B).

**TABLE 2:** Sample tag distribution summary table.

#SampleID	Clean_tags	Valid_tags	Valid_percent (%)	Valid minLength	Valid meanLength	Valid maxLength	OTU_counts	Total OTUs
C1.D8	71,734	65,958	91.95	256	416.09	439	1,594	4,135
C2.D8	74,044	68,076	91.94	228	414.24	455	1949	4,135
C3.D8	41,264	37,203	90.16	229	416.22	441	1,429	4,135
C4.D8	72,438	67,264	92.86	228	412.12	440	1733	4,135
C5.D8	71,160	66,202	93.03	228	414.04	437	1,694	4,135
C6.D8	73,320	67,309	91.80	236	414.18	440	1,661	4,135
C8.D8	73,930	68,725	92.96	228	414.27	440	1753	4,135
M1.D8	71,501	67,026	93.74	228	414.38	441	1,441	4,135
M2.D8	72,059	65,866	91.41	228	417.12	442	1,307	4,135
M3.D8	71,152	67,237	94.50	228	415.51	440	1,257	4,135
M4.D8	73,322	68,919	93.99	259	406.4	446	1,152	4,135
M5.D8	72,402	68,246	94.26	248	412.6	440	1,206	4,135
M6.D8	73,007	69,406	95.07	256	405.99	438	1,099	4,135
M7.D8	73,066	67,713	92.67	258	410.17	441	1,312	4,135
M8.D8	73,764	69,241	93.87	222	409.59	441	1,263	4,135
SA1.D8	72,246	67,236	93.07	228	415.88	450	1,100	4,135
SA2.D8	67,957	58,735	86.43	258	415.37	455	691	4,135
SA3.D8	69,731	62,157	89.14	256	414.68	445	930	4,135
SA4.D8	69,355	61,676	88.93	258	416.79	438	1,413	4,135
SA5.D8	71,149	65,883	92.60	228	412.19	441	889	4,135
SA6.D8	71,172	63,337	88.99	228	416.76	437	1,495	4,135
G1.D8	72,469	66,439	91.68	228	413.08	437	1,379	4,135
G2.D8	71,143	66,258	93.13	248	411.4	440	1,438	4,135
G3.D8	72,253	64,874	89.79	229	412.79	442	1,392	4,135
G4.D8	70,144	64,416	91.83	258	415.05	440	1,091	4,135
G5.D8	70,345	63,676	90.52	228	410.17	441	1,402	4,135
G6.D8	70,384	61,150	86.88	258	410.15	441	1,268	4,135
G7.D8	72,754	68,379	93.99	256	411.65	440	1,291	4,135
G8.D8	70,885	65,241	92.04	228	411.71	441	1,420	4,135
G4.D8	70,144	64,416	91.83	258	415.05	440	1,091	4,135

We further analyzed the community structural distribution and first calculated the relative abundance based on biological taxonomy levels. The differences in abundance between each group at the phylum level are shown in Figure 2C, where we detected the top 30 representative phyla, in which we observed that colitis mice presented a high relative abundance in *Firmicutes* compared to the other three groups. Subsequently, we focused on the difference in abundance at the family and genus levels (Figures 2D,E) in each group. At the phylum level, colitis mice presented a high relative abundance of *Proteobacteria and Firmicutes*, which had been proved to be a signature of dysbiosis of the gut microbiota. Mice treated with ginger and SASP showed completely opposite results. We also observed a slight increase in *Gemmatimonadetes*. At the family level, *Lachnospiraceae* showed a significant increase in mice treated with DSS than in the control group. Ginger and SASP decreased the relative abundance of *Lachnospiraceae*. In contrast, *Muribaculaceae* were reduced in colitis mice, and ginger corrected this disorder. At the genus level, *the Lachnospiraceae_NK4A136_group* showed a sharp increase in the model group. Finally, we calculated the average data in each group and performed a heatmap to illustrate the top 15 different species at the family level (Figure 2F). The heatmap showed that *Lactobacillus_murinus*, *Lachnospiraceae_bacterium_615,* and *Ruminiclostridium_*sp*._KB18* levels increased in the model group but decreased in ginger and SASP treatment groups. However, *Bacteroides_acidifaciens_JCM_10,556* showed an opposite trend.

### The Diversity of Species in the Microbiological Environment

**FIGURE 3:**
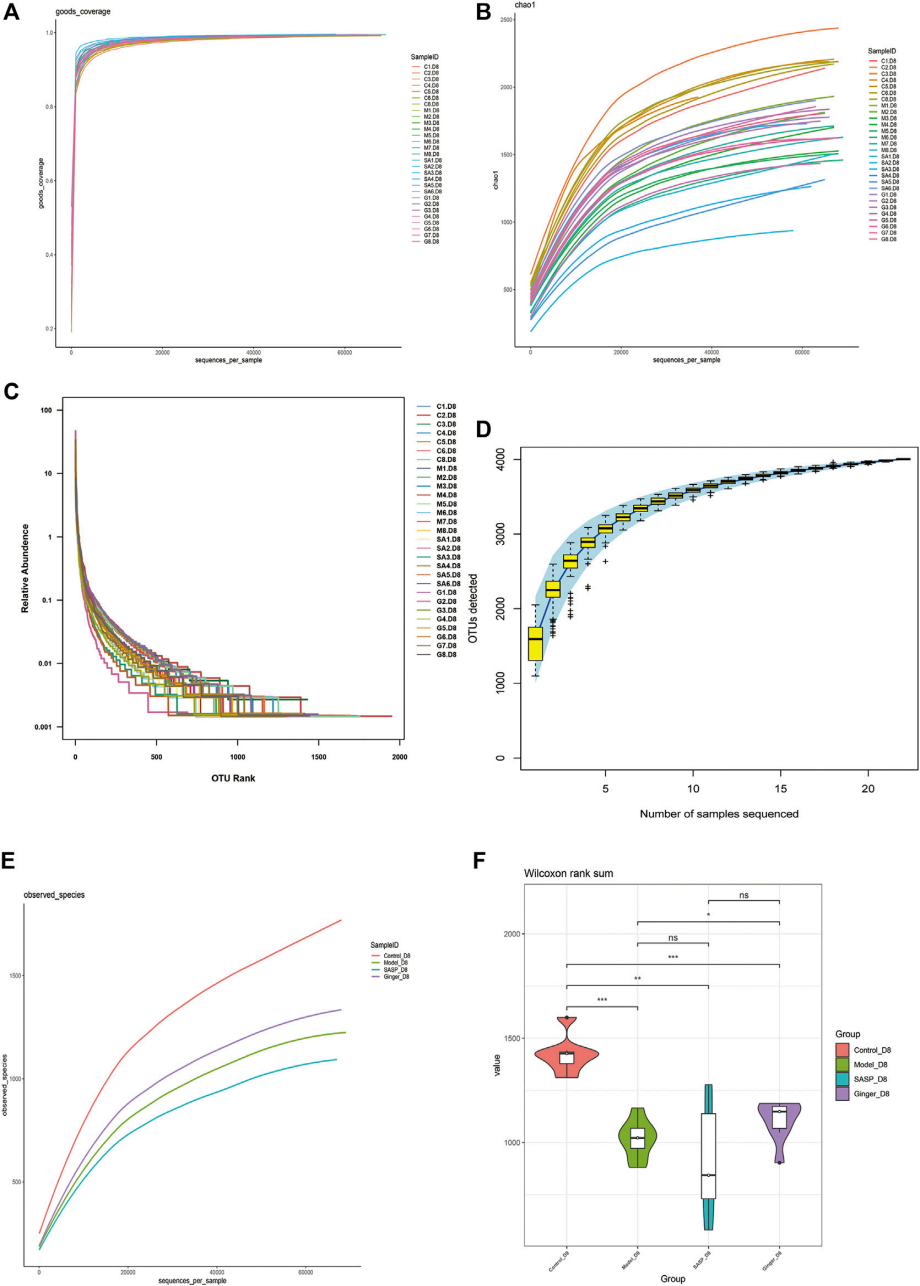
Alpha diversity index calculation statistics correct sequencing depth. **(A)** Goods_coverage analysis of each sample. Each curve represents a sample. The *X*-axis is the depth of random sampling (number of sequences sampled), and the *Y*-axis is the exponential value. An increase in the number of extracted sequences and the gradually flattening curve indicate that the amount of sequencing data is reasonable. **(B)** The chao 1 estimator. The *X*-axis is the depth of random sampling (number of sequences sampled), and the *Y*-axis is the number of OTUs. **(C)** Species richness and evenness of each group. On the *X*-axis, OTUs are sorted according to the number of sequences they contain. For example, “500” represents the 500th most abundant OTU in the sample. On the *Y*-axis, the relative abundance of OTU, such as “0.01”, represents 0.01%; eg, “0.1” stands for 0.1%. **(D)** Species accumulation curve. The *X*-axis represents the number of samples, and the *Y*-axis represents detected operational taxonomic unit (OTU) numbers. The flat curve indicates sufficient sampling. **(E)** The average OTUs numbers of observed species in each group. The *X*-axis is the depth of random sampling (number of sequences sampled), and the *Y*-axis is the exponential value. When the curve tends to be flat, it indicates that the amount of sequencing data is large enough to reflect most of the microbial species information in the sample. **(F)** Violin diagram shows alpha diversity index. The *X*-axis represents different groups are distinguished by different colors, and *Y*-axis is the index value.

Alpha diversity analysis was performed to observe the species diversity in each individual sample. The constructed dilution curve of the diversity index presents the differences in species richness, and the goods_coverage in Figure 3A shows the sequencing depth with an index value close to 1 that proved the rationality of this analysis. The species richness index was calculated with Chao1 (Figure 3B). Mice with colitis showed a lower richness index, which suggested that DSS disrupted the microbiology structure. The administration of ginger and SASP to mice altered this phenomenon. Rank abundance (Figure 3C) demonstrated the species richness and evenness of each group. DSS disrupted the balance in the composition of the gut microbiota. Mice treated with ginger and SASP showed a higher species richness and evenness compared to the model group. Furthermore, the specaccum species accumulation curve illustrated in Figure 3D showed that the number of species increased with an increasing number of samples, and the gentle curve indicates sufficient sampling. We also obtained the average results in each group and finally obtained an observed species curve in Figure 3E to prove the results. Mice in the control group showed the highest diversity in microbiome species, and mice treated with ginger came next. DSS reduced diversity sharply, and it seems SASP could not reverse this. Finally, we performed Wilcoxon rank-sum, and a violin diagram was obtained, which more clearly indicated the microbial diversity in mice from different groups. (Figure 3F). We confirmed that DSS destroyed the microbiology structure in mice colon, and ginger slightly corrected this; however, the influence was not dramatic.

### Sequencing Depth Correlation and Multivariate Statistical Analysis of Microorganisms

**FIGURE 4:**
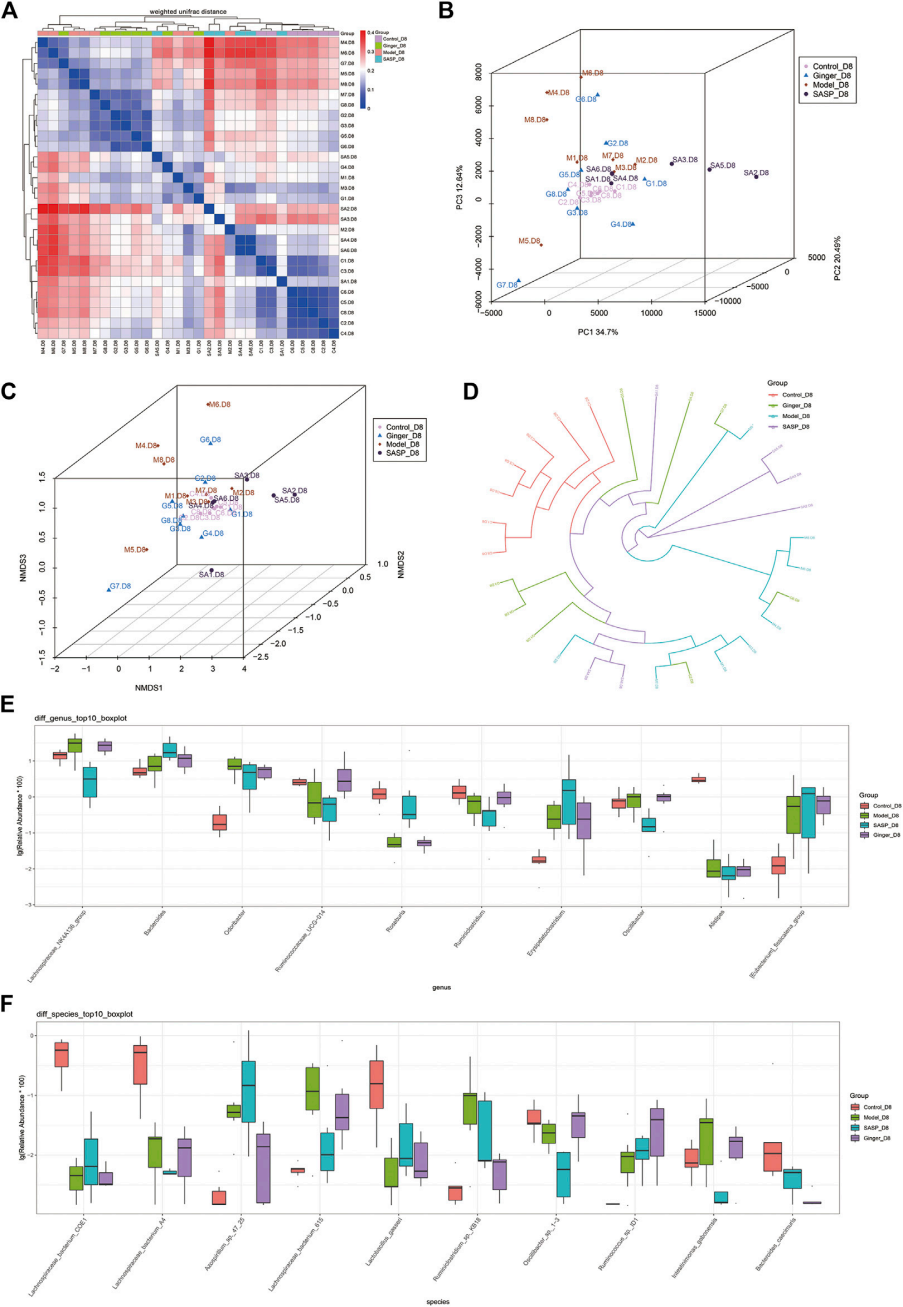
Sequencing depth corrected and multivariate statistical analysis of microorganisms. **(A)** Beta diversity analysis of the variability of each group. In the heatmap (above and left), the clustering branch represents samples from different groups. The deeper the blue, the closer the two groups are. The intensity of the red color indicates the opposite. **(B)** PCoA represents differences between groups. The abscissa (PC1) and the ordinate (PC2) are the two main coordinates with the largest interpretation of the difference between samples. Each point in the figure represents a sample, the same color is the same group, similar samples will be clustered together, if there is a big difference between the samples, the distance will be farther in figure. **(C)** NMDS analysis. Each point in the figure represents a sample, the same color is the same group, similar samples will be clustered together, if there is a big difference between the samples, the distance will be farther in figure. **(D)** UPGMA analysis the differences between samples. Each color represents a group. The closer the branch distance, the more similar the samples. Bar boxes illustrate the relative abundance of dominant species and compared the differences within different groups in **(E)** genus and **(F)** species.

Beta diversity analysis reflects the diversity among habitats, which means the differences between different samples are often based on OTU sequence similarity or the structure of the community (i.e., species abundance and distribution), or both the evolutionary relationship of the OTU sequence and the structure of the community. First, we obtained the beta-diversity index and found that the microbial diversity observed in mice treated with ginger was similar to that of the control group (Figure 4A). We also used principal coordinate analysis (PCoA) of the microbial community to compare the degree of variation between different samples, and the results are shown with a 3D diagram (Figure 4B). Here we conclude that, overall, microbial evolution in mice treated with ginger is closer to that in control mice. A larger difference was observed between saline- and SASP-treated mice with colitis. We observed that these two groups possessed a larger difference compared to the control group. Nonmetric multidimensional scaling (NMDS) is often used to compare differences between sample groups, and it is based on evolutionary relationships or quantitative distance matrices. We also obtained an NMDS 3D diagram (Figure 4C), and the results were consistent with the PCoA. For clarity, we obtained a circular hierarchical clustering_tree (Figure 4D) using the unweighted pair-group method with arithmetic mean (UPGMA) statistics. The distance between the two branches demonstrates the differences between the two samples. Finally, we performed an analysis of variance (ANOVA) and counted the top 10 different abundant microbiomes at the genus level (Figure 4E) and species level (Figure 4F). We found that most of the microbiomes were similar in the other three groups compared to the control group, but there were also some differences in those three groups. At the genus level, SASP and ginger decreased the abundance of *Odoribacter* compared to colitis mice. Ruminococcaceae_UCG-014 was decreased in colitis mice, and SASP did not reverse this, but mice treated with ginger showed a similarity in abundance with control mice. At the species level, we observed a sharp increase in the relative abundance of *Azospiellum_*sp*_47_25* in the model and SASP groups; mice in the ginger group only had a slight increase. We also found that *Lachnospiraceae_bacterium_615, Ruminiclostridum_*sp*._kb 18*, *and intestinimonas_gabonensis* were high in colitis mice compared to control mice, and SASP and ginger reduced this change. Meanwhile, *Lactobacillus_gasseri* and *Oscillibacter_*sp*._1-3* showed contrasting trends.

### Species Correlation and Phylogenetic Analysis

**FIGURE 5:**
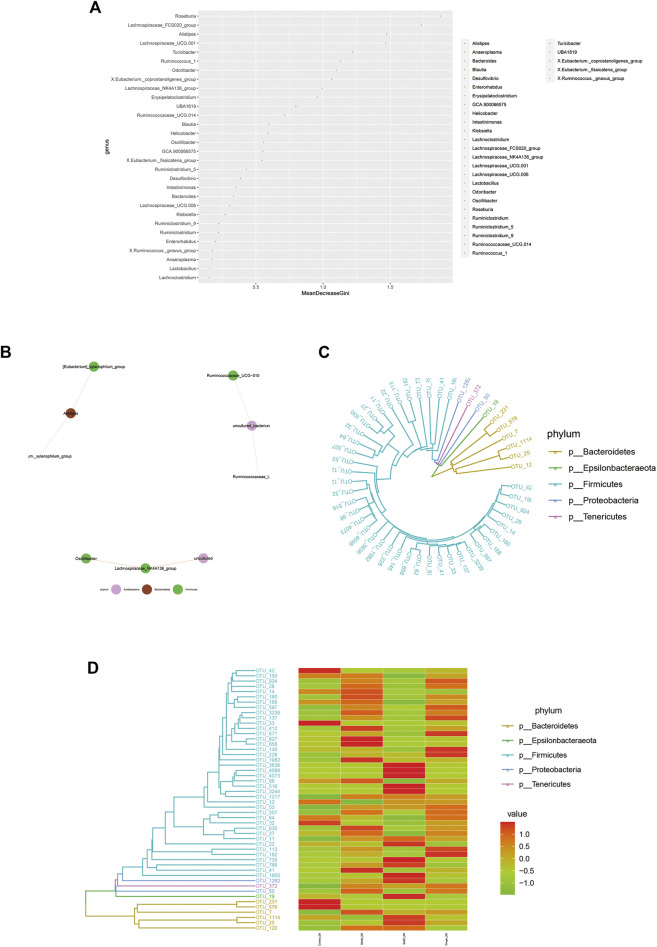
Microbiome community samples classification and phylogeny analysis of phylum. **(A)** Species (variables) importance point map. The abscissa is the importance measurement standard, and the ordinate is the species name sorted by importance. The figure uses standardized importance values by default. **(B)** Species correlation network diagram. The size of the nodes in the figure indicates the abundance of species, and different colors indicate different species; the color of the line indicates positive and negative correlation, red indicates positive correlation, and green indicates negative correlation; the thickness of the line indicates the size of Pearson's correlation coefficient. Thicker line represents more higher the correlation between species. the abundance of OTUs in each sample are shown **(C)** and phylogenetic tree and species abundance combination diagram **(D)** is obtained. The clustering branch represents different bacterial phyla. The abundance graph is shown on the right, which corresponds to the abundance of left operational taxonomic units (OTUs) in each sample.

Random forest is a machine learning algorithm that can effectively and accurately classify microbial community samples and identify different key components (OTUs or species) between regions. We drew a random forest diagram in Figure 5A and predicted the outstanding species. The Spearman correlation coefficient was calculated based on the relative abundance between species samples. The interrelations between species within the sample or group of samples were obtained, and the network of species interactions was constructed using a visual software that showed the interrelations between species. We plotted a network map of species in Figure 5B to exhibit the species correlation of various classification levels under certain environmental conditions. The abundance of each OTU was calculated. TOP50 with the most tags (most abundant) were selected to construct a phylogenetic tree (Figure 5C) and were shown with a heatmap (Figure 5D). We observed a lower abundance of bacteria such as *Epsilonbacteraeota* and *Bacteroidetes* and a higher abundance of bacteria such as *Firmicutes, Proteobacteria,* and *Tenericutes* in colitis mice compared to mice treated with ginger and SASP.

### Gut Microbiome Disorder Contributed to Metabolic Dysfunction

**FIGURE 6:**
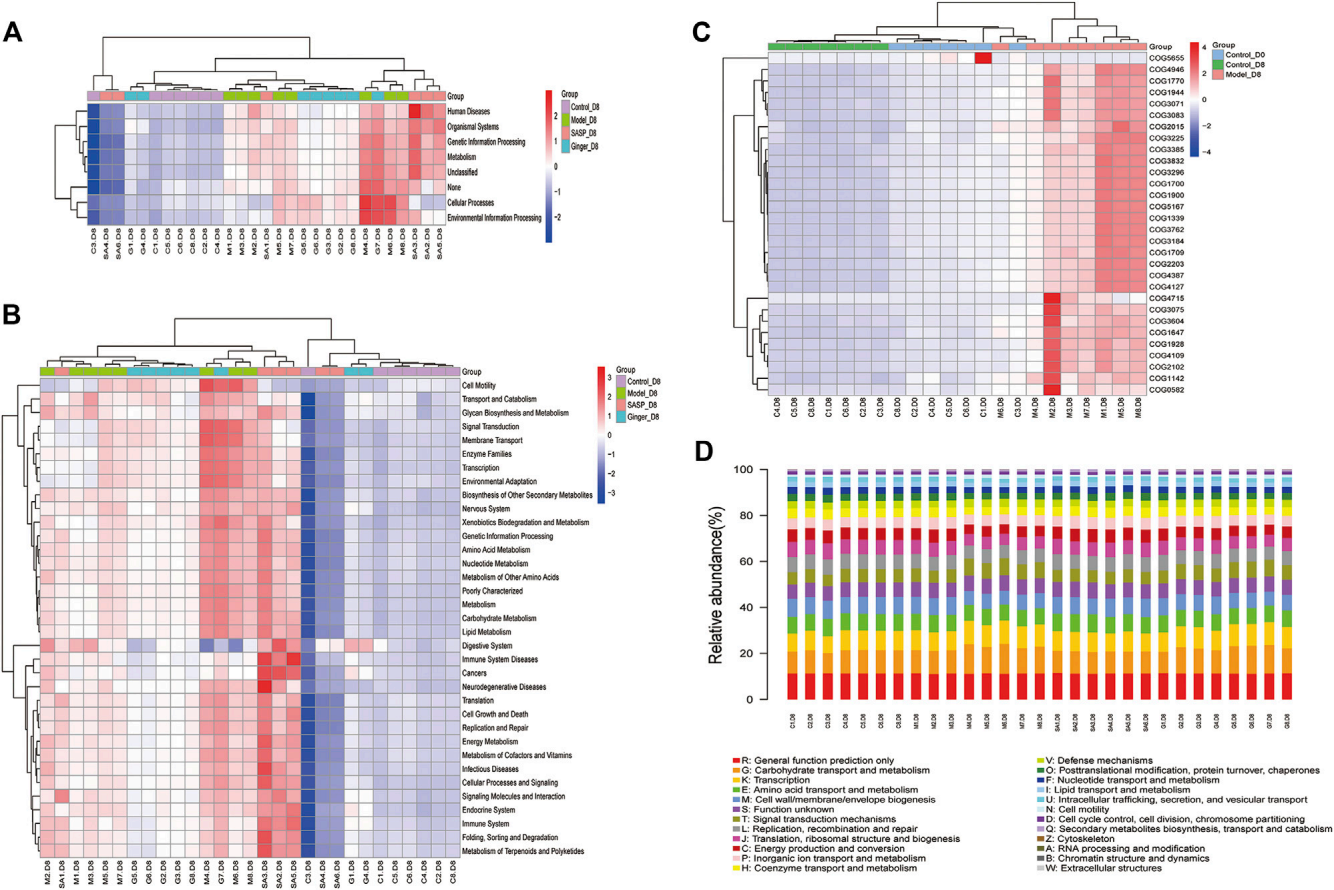
Prediction of the functional composition of the gut microbiome. **(A)** and **(B)** Kruskal–Wallis analysis was performed to predict functions **(A)** and pathways **(B)** the different microbiome species related. The results are shown as a heatmap. **(C)** Clusters of Orthologous Groups of proteins were predicted and shown in the heatmap. The cluster tree on the left represents the clustering of different metabolic pathways, and the above-clustering branch represents samples from different groups **(D)** COG bar plot predicted the microbiome function; the *x*-axis is sample name and the *Y*-axis is relative abundance of predicted COG_category.

As shown in Figure 6A, Wilcoxon analysis was used, and we eventually found that changes in the intestinal flora in different groups showed a close relationship with human physiological function. The most prominent function focused on human disease, organismal systems, genetic information processing, metabolism, and cellular processes. Kruskal–Wallis analysis using the Kyoto Encyclopedia of Genes and Genomes (KEGG) database identified 30 pathways based on different flora (Figure 6B). The predictions revealed that most pathways involved were related to metabolism, including the metabolism of starch, sucrose (and oxidative phosphorylation), and amino acids (alanine, aspartate, and glutamate), as well as genetic information processing (DNA repair and recombination proteins, ribosome, and pyrimidine metabolism). Clusters of Orthologous Groups of proteins (COG) is a database of the NCBI. COG is categorized into two: prokaryotes and eukaryotes. Prokaryotes are generally called COG databases. In this research, cluster analysis of difference results was performed based on COG databases, and the heatmap in Figure 6C shows the top 30 COG related to different species in the four groups. We also generated a bar blot, as shown in Figure 6D, to predict the potential functions microbiomes possess. However, we only found two functions, carbohydrate transport and metabolism and transcription, slightly related to their microbiology.

## Discussion

In this study, we established a DSS-induced mice colitis model to determine whether ginger restored the gut microbiome composition or not. We also chose SASP, a sulfa antimicrobial that has been used as a therapy for IBD (Sutherland and Macdonald, 2006), as a positive control. Our findings revealed that ginger inhibited colitis progression, alleviated colon injury, and regulated the fecal microbiome.

In recent years, many investigators have researched the treatment mechanism of SASP, especially its regulatory effect on the intestinal microbiome. It has also been proven that SASP altered the gut microbiome and restored the TNBS-induced gut dysbiosis in TNBS-induced colitis (Zheng et al., 2017). We have also mentioned that SASP is widely chosen as a positive control in research that elaborates on the regulation effect of rhizome herb on intestinal microbiota in experimental IBD (Liu et al., 2018; Jia et al., 2020). In our study, the results indicated that ginger alleviated weight loss and reduced DAI in colitis mice, an effect that is similar to that of SASP. The results also showed similar effects on the changing of colon length and spleen index. Although ginger was slightly disadvantageous in reducing the expression of *IL-6*, it exhibited the same efficiency in regulating the expression of *iNOS.* Therefore, we proved that oral ginger delayed DSS-induced colitis progression in Balb/C mice.

A community structure or a biological community refers to all creatures with a direct or indirect relationship to each other in the communal biological environment or the collection of all organisms. The various groups in a microbial community coexist and have distinct types of nutrition and metabolism (Hooper et al., 2012). Imbalances in the gut microbiome disrupt homeostasis and contribute to UC; therefore, novel treatment approaches such as manipulation of the gut microbiome (Cohen et al., 2019) and FMT have been proposed for treating UC (de Groot et al., 2017). The microbial diversity results of our study suggested that the ecological structure of the intestinal flora was disrupted as colitis progressed. Our analysis of the community structure revealed a high composition of *Proteobacteria*, *firmicutes*, *Gemmatimonadetes,* and *Lachnospiraceae* in DSS-induced model mice, which was reduced by ginger and SASP. Ginger especially showed a higher efficiency compared to SASP. In contrast, *Muribaculaceae* was decreased in colitis mice. In more detail, we found that *Bacteroides_acidifaciens_JCM_10,556,* a species in the *Bacteroides* phylum, was decreased in the model group; although ginger could also correct this disorder, SASP almost reversed this change.

The presence of *Proteobacteria* is a sign of microbiota disorder, and increased levels contribute to dysbiosis and the risk of disease (Shin et al., 2015). In addition to IBD and other intestinal diseases, asthma and chronic obstructive pulmonary disease have also been observed to be associated with increased *Proteobacteria* composition (Rizzatti et al., 2017). Recent studies have revealed the relationship between a high abundance of firmicutes and IBD risk (Eom et al., 2018). *Gemmatimonadetes*, one of four major families of organisms constituting the human gut microbiota, plays an important role in gastrointestinal and systemic diseases (Binda et al., 2018). Ginger reduced the abundance of these pathogenic bacteria and was an active component that improved colitis via the gut microbe regulation. *Lachnospiraceae* depletion was reported in most research (de Oliveira, 2019), contrasting with our research; this needs more experimentation to explore the changes accurately. *Bacteroides* imbalance was reported in IBD patients who presented with a lower abundance compared to healthy humans (Brown et al., 2019). Bacteroides ovatus monotherapy has also been proven to be more effective than FMT to treat IBD (Ihekweazu et al., 2019). In this study, we also found a decrease in the relative abundance of *Bacteroides_acidifaciens_JCM_10,556* in model groups*.* Ginger and SASP slightly improved the abundance.

Alpha diversity analysis reflects the diversity of species in the biological environment, and Beta diversity is the degree of diversity between habitats, that is, to compare the differences of samples in different groups. Two methods were used in this research. Colitis mice treated with SASP and ginger all showed a relatively healthy gut microbiome status and similar microbial diversity to mice in the control group. According to Alpha analysis results, ginger was more effective than SASP, suggesting that ginger stabilized the gut microbiome. This observation might explain the use of ginger as a traditional medicine for alleviating gastrointestinal diseases, such as IBD and necrotizing enterocolitis (Cakir et al., 2018). However, some microbiomes showed an opposite trend with the control group, and this needs further verification.

The gut microbiome is well known to play a major role in sustaining human health, and temporal and spatial changes in the gut microbiota occur throughout life (Jandhyala et al., 2015). Important metabolic pathways such as lipid metabolism (Wang et al., 2016), drug metabolism and efficacy (Wilson and Nicholson, 2017) and the production of tryptophan, phenylalanine, and tyrosine (Dodd et al., 2017) have been shown to be modulated by the gut microbiome. In this study, we first analyzed the correlation between different diseases and physiological functions. The results showed that the differences between the four groups contribute to human disease, organismal systems, genetic information processing, metabolism, and cellular processes. For further analysis, we used the KEGG database to explore the metabolic changes caused by different gut microbiome structure in colitis mice and identified 42 pathways related to the different microbiome. We found that gut microbiome disorder disrupts metabolic pathways of amino acid metabolism, as well as oxidative phosphorylation and genetic information processing, such as translation and ribosome biogenesis.

We predicted that gut microbiome disorder significantly contributed to the upsetting of metabolic pathways such as transport and catabolism, oxidative phosphorylation, glycan biosynthesis and metabolism, and biosynthesis of other secondary metabolites. Researchers have shown that changes in carbohydrate metabolism and amino acid biosynthesis are related to nutrient transport and uptakee (Morgan et al., 2012). Epidemiological research has also shown that antibiotics over-scavenge the gut flora, causing macrophages to overreact to bacteria, thereby disrupting oxidative phosphorylation. Hypermutation occurs in 27% of IBD-associated colorectal cancer cases, including DNA repair dysfunction (Din et al., 2018). *Lipopolysaccharide* isolated from *Helicobacter* disrupts intestinal DNA repair, increasing the risk of permanent genotoxic effects, contributing to IBD or colon cancer (Cavallo et al., 2011). The COG prediction results also focus on nucleic acid and metabolism-related functions. We observed a significant relationship between the different species and RNA processing and modification, chromatin structure and dynamics, amino acid transport and metabolism, and nucleotide transport and metabolism.

In conclusion, the results in this research showed that ginger ameliorates the severity of DSS-induced colitis in mice. We also observed that ginger treatment altered the intestinal microbiomes of colitis mice, increasing the relative abundance of some species. According to KEGG and COG analysis, the observed changes have a close relationship with the physiological functions of mice. Therefore, we hypothesize that the IBD alleviation effect of ginger may be related to the function of intestinal bacteria. However, the components of ginger that contribute to its regulatory effects on the gut microbiota and mediate its therapeutic effects still need further exploration.

## Data Availability

The data presented in this article are deposited in the NCBI Sequence Read Archive repository, accession number PRJNA680889.
